# Unauthorized Renewal of Prescriptions

**Published:** 1869-03

**Authors:** 


### Editorial Department.

### Unauthorized Renewal of Prescriptions.

During the past few months the questions of the right and the safety and propriety of druggists renewing prescriptions without the consent or knowledge of the prescriber, has been extensively discussed in the medical societies and journals of the country, and legislative action has been proposed with the view of correcting the evil. We do not propose to discuss the general questions at issue, but shall assume that druggists have, at least, no moral right, and that patients would hardly ever, if ever, desire the renewal of a prescription in opposition to the advice of the prescriber. There are instances, where prescriptions are renewed for years, are retained and duplicated and distributed promiscuously through neighborhoods and circles, and made to answer an extensive round of practice, but in the great majority of cases they are used legitimately and fairly. It is with no mercenary intentions that physicians object to unauthorized renewal of prescriptions, though it may be confessed that oftentimes, and especially in the treatment of certain classes of specific diseases, it is a great abuse, depriving the physician of his pecuniary recompense, and at the same time rarely advantageous to the patient. The practice of renewal has grown out of conditions which it is now not so easy to control. In the first place, some patients regard the prescription as their property—as the consideration they have received for their money, and insist upon unlimited control of it; whereas it is really only a private note to the druggist, giving directions to him how to prepare the medicine they are advised to use. But it is not so regarded by many patients. They consult physicians who always give written prescriptions, rather than furnish remedies, because they consider it a standing advice to be used under similar circumstances forever. It is a part of the expectation, and whether justly founded or not, it requires time and judicious management to correct it; but it can be controlled and modified, and in time wholly changed. Again, the druggist has some interests in the premises which are to be considered, though the physician and druggist are nearly related in the treatment of disease, and their interests are not conflicting. Renewal is not more profitable than first compounding, but the druggist when once in possession of prescriptions controls the place of purchase; patients can obtain the medicine of them, and nowhere else. This is the only motive, so far as the interest of the druggist appears, and in this it is only conditional and by consent, for prescriptions are returned or copies are given whenever asked for as an almost universal rule. Patients can thus continue the treatment advised as long as they please, without renewing their advice, giving the physician less opportunity to "watch the case" and renew the fee. To this we do not object, so far as the physician is concerned, or the patient, if it is followed within the bounds of safety and judicious care. But how many of the prescriptions for acute diseases are unsafe to renew; how frequently we write for medicines which are perfectly safe and harmless in the quantity prescribed, but in duplicate would prove injurious and even quite unsafe?

All physicians write some prescriptions which are to be renewed, which it is a great convenience to have renewed; all write some which cannot be renewed and continued indefinitely without injury and risk. It requires no argument to show these obvious facts, and it seems to us that it requires no legislation to correct the evil. When we make prescription we write directions for administration--write the quantity and the frequency. If the medicine may be continued and renewed we may write, to be renewed; or, if otherwise, not to be renewed. No respectable druggist will disregard this simple request. The druggist will, of course, inform the applicant that his physician advises discontinuance of the remedy without further instructions, and thus all the common dangers and objections to renewal will be obviated. Every one will earnestly and heartily second this much of the plan, since all understand that the most simple and harmless remedies, when properly prescribed, become injurious in the highest degree by improper continuance. There are prescriptions which are given for chronic and specific diseases which are not easily brought under control, in which our directions will not be regarded, and may not perhaps with safety be made known. In such case private advice or direction to the druggist would be safer and better.

The general custom of renewing prescriptions has grown up with us by the mutual consent of all parties, and is not more the fault of the druggist, not so much the fault of the druggist as of the physician, if it may be regarded as from fault in either. Physicians have never written any directions to the druggist in regard to the continuance of remedies, and all instruction concerning this matter has been vested in the patient. Such being the case, it has been all right to renew as often and as long as desired; to sell the compound in greater or less quantities to suit the wishes of purchasers, or if not all right, at least all very natural and excusable in the druggist.

Physicians, we believe, hold this whole matter in their own hands. If it is an abuse, and there is no doubt it is, it is the fault thus far in our history, of the prescriber and not of the compounder of prescriptions. It has been an universal practice, receiving the consent, and we add, sanction of physicians.

What shall be done to correct the custom and insure the proper uses, without any of the abuses which are liable to be made of the advice we give for the care of the sick? All prescriptions, not designed for renewal, should be marked as improper for repetition, and this should constitute a part of the direction in connection with the dose and frequency of administration.

Will any druggist, who desires to compound the prescriptions of physicians, disregard this part of the direction any more than that concerning the dose and frequency? Does it require any other law than the natural one of self-defence, to insure a faithful adherence to such instructions? We believe that the united and harmonious action of the profession will regulate this question upon which so much has been said, with the unanimous consent and perfect satisfaction of all parties. If not, physicians can go back to the former practice of compounding their own prescriptions, a habit which has never been abandoned by some, mainly because of the impositions practiced upon them by improper use of their written directions for treatment of specific and some forms of chronic diseases. We shall strongly incline to the adoption of this method if we find it impossible to control in this important and reasonable action. Speaking for the druggists of Buffalo, we believe they enjoy the confidence of the profession in such degree that they will be found ready to adopt any measures of reform that may be agreed upon by physicians. It is objected, that some druggists would readily and cheerfully second such reform, while others would continue the plan of renewal. Possibly this might be so, but we should soon see how much influence physicians have over the place and manner in which their prescriptions are compounded.

This subject will come up for action in the Buffalo Medical Association. Harmony of purpose and action will insure complete success.

